# Response to Rituximab in a Case of Lupus Associated Digital Ischemia

**DOI:** 10.1155/2014/763608

**Published:** 2014-07-09

**Authors:** Orhan Küçükşahin, Nurşen Düzgün, Alexis K. Okoh, Emre Kulahçioglu

**Affiliations:** ^1^Department of Rheumatology, Ankara University Medical Faculty, Sihhiye, 06410 Ankara, Turkey; ^2^Department of Internal Medicine, Ankara University Medical Faculty, Sihhiye, 06410 Ankara, Turkey

## Abstract

We report the case of a 38-year-old female patient with systemic lupus erythematosus (SLE) and Jaccoud arthritis (JA) that sequentially developed digital ischemic lesions of the hands. In spite of follow-up treatment with glucocorticoids, immunosuppressant, antiaggregant, and potent vasodilatator agents, a serious progression to digital gangrene over a one-month period was observed. Surprisingly, her nonhealing digital lesions improved after two cycles of rituximab (RTX) administration.

## 1. Introduction

Systemic lupus erythematosus (SLE) is a multisystemic autoimmune disease that is associated with considerable morbidity and mortality. Digital ischemia, digital ulcer, or gangrenous lesions have been described in SLE [1, 2]. Vascular damage in SLE most likely occurs due to vasculitis, premature atherosclerosis, and hypercoagulability related to antiphospholipid antibodies. Vasculitic ulcers tend to be chronic if not well treated and may lead to significant impact on the psychosocial as well as physical well-being of the individual. The gangrene could also contribute to other conditions such as anatomical changes [1].

Presented here is an SLE-JA patient with subluxation of MCPs swan neck deformities who sequentially developed digital ischemic lesions after stopping immunosuppressive treatment at her own discretion due to exorbitant side effects. Digital ischemia of hands showed a serious progression to digital gangrene over a month in spite of follow-up treatment with glucocorticoids, immunosuppressant, antiaggregant, and potent vasodilatator agents.

Surprisingly, her nonhealing ulcers improved dramatically after two cycles of rituximab RTX administration. To the best of our knowledge, this is the first SLE-JA case presenting with nonhealing digital ischemic lesions responding dramatically to RTX therapy in the medical literature.

## 2. Case Presentation

A 38-year-old Turkish woman with a history of SLE presented in 2003 with photosensitive rash on her face and chest, oral aphthous lesions, and arthritis with pain and motion restriction in hands, wrists, elbows, ankles, and knees. A diagnosis of SLE associated arthritis was made and treatment with 5 mg of prednisone once daily and 200 mg of hydroxychloroquine twice daily was started requiring the addition of methotrexate in escalation until 25 mg weekly was reached. Her joint manifestations were partially responsive; however, arthritis remained active in the small joints of her hands with pain and swelling. Six years later, the patient developed avascular necrosis during follow-up for which she was operated on at an outside setting and a hip prosthesis was implanted.

**Figure 1:**
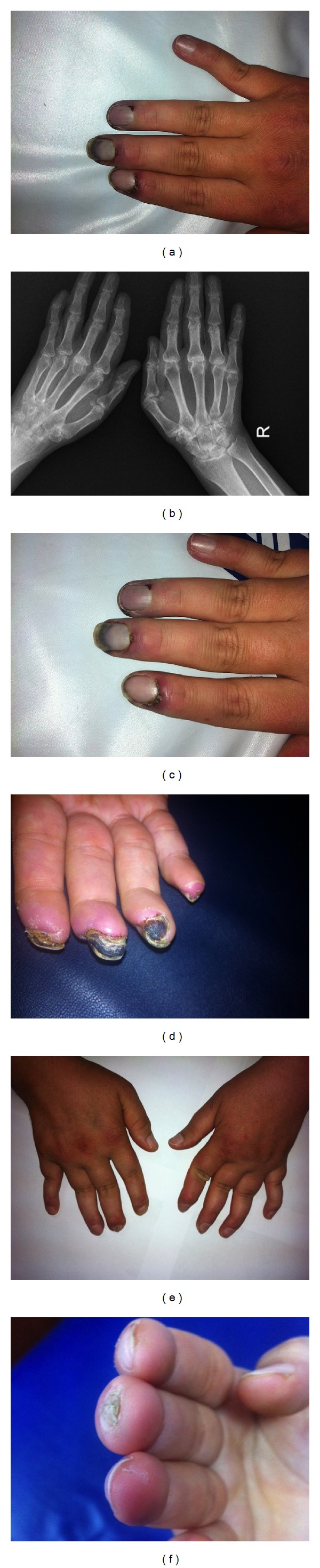
(a) Right hand with swan neck and Z deformity of the thumb (before treatment). (b) Radiograph; AP of bilateral hands suggesting Jaccoud radiological features. (c) Digital ulcers of the second to fourth digits of the right hand (2 weeks after initial treatment with cyclophosphamide). (d) Unresponsiveness to initial treatment demonstrated by a serious progression from ischemic lesions to digital gangrene lesions of the second to fourth digits of the right hand (one month after initial treatment regimen; cyclophosphamide, corticosteroids, and prostaglandins). (e) Response to treatment observed after first cycle of rituximab therapy (5 months following the start of RTX treatment). (f) Complete remission of digital gangrenous lesions after 2 cycles of rituximab therapy (10th month of the RTX treatment protocol (rituximab, glucocorticoid, aspirin, azathioprine, and hydroxychloroquine)).

Two years following surgical treatment for avascular necrosis, the patient presented to our rheumatologic polyclinic with complaints of pain, swelling, warmth, and cyanosis over the fingers of both hand and toe tips. Physical examination revealed swelling, warmth, and tenderness in the proximal interphalangeal (PIP) joints of both hands, Ulnar deviation, and bilateral swan neck deformities. Right is greater than left. “Z” deformities of thumbs (Figure 1(a)) and cyanotic ulcers were seen on the third to fifth digits (Figure 1(c)) and on both digits of feet. All described deformities were reducible. Peripheral pulses were normal. In addition, she had a history of photosensitivity and two gestations with two uneventful births. On further interrogation, the patient was admitted to stopping medical treatment at her own discretion three years before presentation due to exorbitant side effects.

Laboratory findings were as follows: WBC: 5.6 × 109/L, Hb: 11.9 g/dL, platelet: 276 × 109/L, Hct: 35.4%, ESR: 120 mm/h, CRP: 80 mg/L, microscopic hematuria and trace proteinuria on urinalysis.

Immunologic studies revealed an ANA titer of 1 : 3200, (++++, speckled pattern), U1RNP (+++), p-ANCA (+), antibeta 2 GP1-IgA isotype (43,5 U/mL; normal: <5 U/mL). There was hypocomplementemia (C3: 0,647 g/L, C4: 0,0758 g/L; normal: 0.9–2 g/L and 0.1–0.4 g/L, respectively.). ds-DNA and Sm antibodies and rheumatoid factor were negative.

Review of hand radiographs showed no erosive changes consistent with a diagnosis of JA (Figure 1(b)). Electrocardiographic, echocardiographic, and computed tomography (CT) imaging studies of the thorax did not show any pathological findings.

Results from laboratory studies showed high disease activity of lupus. Initial treatment was started with glucocorticoid (1 mg/kg/day), aspirin (300 mg/day), IV prostaglandin (2 mcg/kg/min), and IV cyclophosphamide (500 mg/m^2^).

Unexpectedly, digital ischemia of hands showed a serious progression to digital gangrene (Figure 1(d)) over a one-month period in spite of treatment with glucocorticoids, immunosuppressant, antiaggregant, and potent vasodilatator agents.

Following the first month of unsuccessful treatment with the initial treatment regimen, a new treatment protocol including rituximab (RTX) with 2 infusions of 1 g at a 14-day interval in combination with hydroxychloroquine and aspirin was initiated during the second month. Clinical and laboratory response to treatment began to be observed during the third month of RTX therapy. Laboratory evaluation revealed the following: ESR: 22, CRP: 3 mg/dL, and normal levels of C3 and C4 complement levels. (Results from a complete blood count panel were unremarkable). A combining dosage of 100 mg/day azathioprine (AZA) was added to the treatment protocol after two infusions of RTX therapy. Also, corticosteroid dosage which was started at 1 mg/kg was tapered gradually by 10% per kilogram for every 10 days till it reached 5 mg/day over the three-month period.

Eventually, digital gangrenous lesions started to regress after the first cycle (5 months) of RTX therapy (Figure 1(e)). A second cycle of RTX therapy was started during which a complete recovery of digital lesions (Figure 1(f)) and the regression of active disease signs and acute phase responses were seen. The patient has been in clinical remission with glucocorticoid (5 mg/day), aspirin (100 mg/day), azathioprine (2.5 mg/kg/day), and hydroxychloroquine (200 mg/day) for the last 2 years.

## 3. Discussion

Vasculitis and digital gangrene have been described in SLE. Thromboembolism, premature atherosclerosis, overlap syndrome, and especially antiphospholipid antibodies (aPLs) may contribute to the development of gangrene [3, 4]. In a study of 485 SLE patients, critical peripheral ischemia was observed in 7 patients (1.4%). 4 of the 7 patients had a positive aPl/LAC [5]. Liu et al. found that 18 of 2684 SLE patients had digital gangrene and Raynaud phenomenon, the long disease duration and elevated serum CRP may be predictive factors for SLE patients to develop the digital gangrene [3].

The prevalence of digital gangrene in APS patients has been reported to be between 3.3 and 7.5% and the presence of a positive anti-RNP has been described in some APS digital ulcer cases [6]. The relationship between a positive anti-RNP and Raynaud phenomenon has been shown [7, 8] and thought to play a role in the development of digital gangrene. To date, the exact etiology of SLE digital gangrene remains unclear and complex with the presence of APS, overlap syndrome, atherosclerosis, or vasculitis appearing as potential causes. A complication of APS is seen as the most probable cause. In our patient, active lupus disease, positive RNP, and antiphospholipid antibodies may play a role in the development of digital vascular lesions.

RTX has been shown to be effective in the reduction of B-cell production and used in the treatment of refractory SLE or vasculitis. In a prospective study conducted by Smith et al., rituximab was shown to play a role in B-cell depletion offering the prospect of sustained disease remission and improved disease control with low toxicity in patients with active refractory SLE or ANCA associated vasculitis (AAV) [9].

In the presence of Apl/LAC, patients were first anticoagulated with IV heparin or low molecular weight heparin (LMWH) before long-term treatment with warfarin [5]. Clinical response was not seen in one of the seven patients and a RTX dosage of 1gram/day was started. An IV dosage of 500 mg cyclophosphamide was added to his treatment regimen during follow-up. An improvement in lesions on day 20 after the first dose with RTX was not recorded [10]. In another case series a low dose of Epoprostenol (0.5 ng/kg/min) was used in the treatment of SLE patients presenting with peripheral ischemic findings and its efficacy was found to be the same as that of higher doses with lesser side effects [5, 11]. Epoprostenol has been shown to be useful in the treatment of necrotic digital ulcers secondary to APS [12]. In the treatment of patients with severe ischemia, a combination with LMWH is preferred. It is described to be effective against microvascular thrombotic occlusions due to its anticoagulant properties [10].

In our patient, digital ischemic lesions were seen two years after stopping treatment at her own will. We added a treatment regimen of Iloprost and acetyl salicylic acid to her traditional immunosuppressive agents which included steroids, hydroxychloroquine, and cyclophosphamides. Her nonhealing ulcers however prompted us to start an infusion dosage of 1000 mg rituximab (per 0–15 days) therapy considering its role in B-cell depletion and low toxicity in patients with ischemic lesions. Surprisingly, her digital ulcers responded dramatically to rituximab therapy after 2 cycles and a recession in acute phase markers was observed.

To the best of our knowledge, our case is the first case in the medical literature reporting an SLE-JA association developing digital ischemic lesions as a late clinical complication and responding dramatically to rituximab therapy.

As a conclusion in lupus patients with arthritis associated nonhealing digital ulcers under traditional immunosuppressive agents, RTX can be a treatment option.
